# Essential Trace Elements in the Human Metabolism

**DOI:** 10.3390/biology13110908

**Published:** 2024-11-07

**Authors:** José Armando L. da Silva

**Affiliations:** Centro de Química Estrutural, Institute of Molecular Sciences, Departamento de Engenharia Química, Instituto Superior Técnico, Universidade de Lisboa, Av. Rovisco Pais, 1049-001 Lisboa, Portugal; pcd1950@tecnico.ulisboa.pt

A few trace elements are absolutely essential for the human metabolism, despite their low levels in the organism. However, our knowledge about this subject is very limited, and new developments in their metabolic roles require contributions and have consequences in different fields, such as nutrition sciences, medicine, pharmacology, biological sciences, agricultural sciences, and chemistry (with special emphasis on analytical chemistry as a basis for detecting low contents of chemical elements in different types of samples). This Special Issue covers diverse topics related to essential trace elements in the human metabolism, contributing to promoting rich and varied information that is accessible to other researchers, which is facilitated by the fact *Biology* is an open access journal.

It should be noted that the studies on the essentiality of trace elements had important developments during the last century, but these systems were mainly based on the effects on the organism, but usually without detailed information about their actuation. Consequently, additional data are necessary related to the chemical characteristics of each trace element, such as the oxidation state of active species, interferences by other chemical elements, and the presence of organic molecules (in this case, the complexation of some metal ions), since different species can reduce their availability or can promote inadequate metabolic responses. On the other hand, studies on the roles of these chemical elements are not usually applied directly to humans due to the complexity of the system and for ethical reasons; consequently, in order to obtain relevant information, suitable models have been proposed. These models can be employed to specific organs or cells or to animals; however, an oversimplified generalization to humans can lead to inadequate conclusions. Another methodology is the application of surveys to specific groups, but some conclusions can be affected by the subjectivity of the participant responses. Experimental work based on different procedures is an important strategy to achieve significant advances, and review articles are also relevant because they allow systematization, which can stimulate new approaches for further experiments. Both types of articles are in this Special Issue, which compiles six papers [1,2,3,4,5,6] (a majority based on experimental research and also two reviews). It gathers contributions from researchers with different backgrounds, from three continents, and six different countries.

All the essential trace elements proposed in this Special Issue are included in at least one article, with the exception of chromium. This element is associated with the human metabolism of glucose by a metallobiomolecule named chromodulin, but in the last 15 years only three articles (in Web of Science) [7] were published about this topic, and all are not directly related to human metabolism. On the other hand, searching in the same database (Chromium AND “Human Metabolism”) leads to a few more published articles, but chromium is a very marginal topic and not directly related to human metabolism. Consequently, it was not possible to find authors for an article on this topic.

The articles published in this Special Issue are on different themes and cover the effect of the essential trace elements in diverse aspects of human metabolism. The main contributions of all papers published are summarized in the paragraphs below. They appear in the same order as on the webpage of the journal for this issue [8].

Studies on the inadequate regulation of glucose levels can be related to diabetes, a disease widely spread over the world. Trace elements are involved in the metabolism of glucose, as is the case of iodine. It can promote glucose uptake; however, at higher levels of this trace element, it becomes cytotoxic. These studies were carried out with adipocytes and pancreatic beta cells, but additional research using animals can reveal more detailed information about the role of iodine in the metabolism of glucose [1].

On the other hand, research on prenatal nutrition involving some essential trace elements (cobalt, copper, manganese, molybdenum, and zinc) clarifies their importance for the healthy development of future human beings. Studies on the urinary levels of these trace elements crossing with maternal data analyzed by mathematical tools are relevant to defining adequate public health policies, promoting a better quality of life for new generations [2].

Brain manganese accumulation (causing toxic effects) is associated with acquired hepatocerebral degeneration disease. Patients with this disease develop chronic liver sickness. Liver transplantation is currently the most efficient procedure for reducing the level of the degeneration caused by this illness. The determination of the blood levels of essential trace elements (cobalt, copper, iodine, manganese, molybdenum, selenium, and zinc), other essential and non-essential chemical elements, and the relations between the levels of chemical elements and clinical alterations after liver transplantation was carried out. Other trace elements than manganese can also be related to physiopathology and clinical manifestations associated with this disease due to oxidative stress and inflammation in cerebral tissues. Based on these results, it can be helpful to improve the quality of life of patients after liver transplantation. However, the metabolic activity of the trace elements is not yet fully clarified [3].

Cobalt in biological systems has two types of coordination to the metal site: directly coordinated to amino acid residues or as a component of chemical species related to vitamin B_12_ (its biosynthesis is only carried out by bacteria, i.e., humans have no capacity for its synthesis). This metal in humans has a limited number of roles, but its deficiency as well as its excess cause several health problems. Current cobalt applications (including some for medical purposes), derived from anthropogenic activities, provoke alterations in the natural cycles of this metal, which can be toxic for humans. Strategies to reduce its toxicity have been developed, reducing the hazardous effects of cobalt and limiting its spread through the environment, e.g., by its chelation or with technologies allowing the accumulation of cobalt by plants [4].

Zinc is fundamental in diverse steps of human metabolism, and it is associated with immunological responses and viral infections. As a strategy to improve zinc responses, the fortification (by chelating this metal with biomolecules or by its micro- or nanoencapsulation in suitable delivery systems) of food with this essential trace element is a way to increase zinc bioavailability, reducing side effects caused by its deficiency, and enhancing the metabolic responses of organisms against exogenous harmful agents [5].

Coronary artery disease is an important cause for mortality. Zinc and copper deficiency are involved in atheromatous plaque formation, a step to triggering the abovementioned disease. The correlation between these disease levels was evaluated with the SYNTAX (Surgery for the Treatment of Narrowed Arteries) score (algorithm applied to data from coronary angiogram images) with copper and zinc content in hair samples. A significant inverse correlation between disease relevance and copper levels and the copper/zinc in hair samples was observed; however, no significant correlation was determined for zinc content. Hair samples might help us learn more about how both copper and zinc affect coronary artery disease [6]. Curiously, several copper enzymes are related to oxygen species, which are associated with coronary artery disease [9]. Is it a coincidence?

In conclusion, these articles provide the scientific community with new insights with potential application to some topics related to human metabolism. With their availability, these themes can be related to others or inspire new strategies about these subjects. I hope that this information can be valuable for the readers, that they will enjoy it, and that it will provide them with the basis for fruitful ideas, as I had such experiences as the Guest Editor of this Special Issue.
